# The Effect of One-Step or Two-Step MTA Plug and Tooth Apical Width on Coronal Leakage in Open Apex Teeth

**Published:** 2012-03-01

**Authors:** Zohreh Khalilak, Tabasom Vali, Farzad Danesh, Mehdi Vatanpour

**Affiliations:** 1. Department of Endodontics, Dental Branch, Islamic Azad University, Tehran, Iran; 2. Private Practice, Tehran, Iran

**Keywords:** Anterior Teeth, Apexification, Apical Foramen, Dental Leakage, MTA

## Abstract

**Introduction:**

Mineral trioxide aggregate (MTA) has been suggested as an effective material for apical barrier, forming an effective seal against bacterial leakage in teeth with open apices. MTA needs moisture for setting; which can come from the apical region or a moist cotton pellet. This study was intended to compare bacterial leakage in one- and two-step MTA apical barrier technique in open apices with different diameters.

**Materials And Methods:**

The root canals of 52 extracted human maxillary incisors were prepared and open apices in two different diameters of 1 and 1.4 mm were created. The samples put in experimental groups randomly. A 4-mm thickness of MTA was placed as apical barrier for both one- and two-step methods. In one-step groups (1.4 mm diameter; n=12 and 1 mm diameter; n=12), the samples were obturated immediately after placing MTA plug. For two-step groups (1.4 mm diameter; n=12 and 1 mm diameter; n=12), a moist cotton pellet was placed over the MTA plug for 3 days before root canal obturation. Four samples served as positive/negative control groups. After one week, microleakage was evaluated using bacterial penetration technique and results were statistically analyzed utilizing SPSS software and Chi-square test.

**Results:**

In one-step technique 13 and in two-step technique 12 samples showed bacterial leakage. There was no statistically significant differences between two techniques (Chi square, P=1). The difference between the results related to 1 and 1.4 mm apical foramens was also not significant (P=1). Also 12 and 13 samples showed bacterial contamination in teeth with 1 mm and 1.4 mm apical foramens, respectively (P=1).

**Conclusion:**

It seems that leakage of MTA apical plug using one- and two-step technique is comparable; however, in vivo investigations are highly recommended for more accurate results.

## Introduction

One of the treatment challenges of immature pulpless teeth is lack of apical stop for sealing the apex prior to root canal filling [1]. In addition, thin and fragile dentinal walls make it difficult to achieve an apical seal [2]. Lack of apical stop and extrusion of filling material through the canal may result in apical leakage [1]. Materials which were previously considered as barriers include calcium hydroxide, tricalcium phosphate, dentin chips, and freeze-dried cortical bone/dentin [3][4][5][6][7].

Apexification is a traditional treatment of these teeth but long term calcium hydroxide therapy has some disadvantages. This treatment requires high compliance of patients and multiple appointments. Thin and fragile dentinal walls make the teeth susceptible to fracture [1][2][7][8]. Mineral trioxide aggregate (MTA) has been advocated as an apical barrier [9]. This material holds promise because of its sealing capabilities [10][11][12][13], setting in presence of blood, and its biocompatibility [14][15][16][17][18][19][20][21].

Various investigations have been conducted on treatment of open apices teeth with MTA plug [22][23]. MTA is a hydrophilic material; hence, it is highly recommended to place a moist cotton pellet over the MTA [2]. In one-step apical barrier technique it is not possible to place a moist cotton pellet over the MTA; this may adversely affect the optimal setting of MTA and its physical properties such as microleakage [23]. Thus, the question about the necessity of using moist cotton pellet over the MTA as apical barrier is still remained. With open apex, MTA may set in the presence of periapical moisture and if this is true, the material could be used in a one-visit procedure. Matt et al. demonstrated that two-step technique showed significantly less dye leakage than the one-step technique of barrier placement [23]. AL-Kahtani et al. showed that there is no difference between one- and two-step apical barrier technique against the bacterial leakage; however, two-step application failed to provide adequate sealing [22].

Many in vitro methods have been used to evaluate the sealing ability of root canal filing materials by using dyes, radioisotopes, scanning electron microscopy, fluid filtration techniques, electrochemical methods, and bacteria/endotoxin. Among these techniques, the bacterial leakage model is considered to be the most clinically relevant [24].

The purpose of this in vitro study was to investigate and compare the effect of one- and two-step MTA barrier technique on apical leakage in two different apical diameters.

## Materials and methods

We used 52 extracted, human, single rooted, maxillary incisors for this study according to perform pilot study. The teeth were stored in 5.25% NaOCl for 1 hour [18]. The teeth were decoronated with 0.3 diamond disc (Drendel and Zweiling Diamant GmbH, Lemgo, Germany) so that the roots were adjusted to approximately 15 mm. Approximately 2 mm of the apical tip of the root was resected with a #245 fissure bur (Drendel and Zweiling Diamant GmbH, Lemgo, Germany) to remove any apical deltas and standardize the canal exit to the center of the tooth [22].

The standardized 13 mm root lengths were prepared to simulate the clinical situation of an open apex. Samples were divided into four groups by random number table. Working length was determined visually 0.5 mm shorter than the apex with stainless steel files #15 (Dentsply, Maillefer, Tulsa, OK, USA). For the first two groups, the canals of 24 experimental teeth and 2 control teeth were instrumented with #1 peeso reamer (Dentsply, Maillefer, Tulsa, Ok, USA) up to the working length. A divergent open apex was prepared by retrograde apical preparation with #40 race .010 taper (FKG Dentaire, Ballaiques, Switzerland) inserted to the length of 6 mm of the cutting blade (D=6 mm) to obtain the 1 mm apical diameter. Then these 24 samples were divided into two groups (one- and two-step MTA application) the canals of other 24 experimental teeth and 2 control teeth were instrumented with #3 peeso reamer to the working length and a divergent open apex was prepared by retrograde apical preparation with a #40 Race. 010 taper inserted to the length of the cutting blade (D=10) to obtain the 1.4 mm apical diameter. Throughout instrumentation, 5.25% NaOCl was used for Irrigation. Blunderbuss shape of the apices confirmed with radiographs. The samples stored in saline.

Then the roots, from the apex to the CEJ, mounted into wet flower arrangement foam. The moist cotton pellet was placed into the foam over the open apices. MTA (ProRoot; Dentsply, Tulsa Dental, Tulsa, Ok, USA) was mixed according to the manufacturer’s instructions. We used a messing gun (Dentsply, Maillefer, Ballaigues, Switzerland) to place the MTA as close to the apex as possible. Hand plugger (Dentsply, Maillefer, Tulsa, Ok, USA) and the thick end of moistened paper points were used to condense the MTA to the apex. Radiographs were taken to ensure proper placement and 4 mm thickness. Any residual MTA on the canal walls coronal to the 4 mm barrier was removed with moistened paper points and #50 stainless steel hand file.

In one-step groups (12 samples with 1.4 mm apical diameter and 12 samples with 1 mm apical diameter), the samples were obturated without delay after application of MTA with AH26 sealer (Dentsply, DeTrey GmbH, Konstanz, Germany) and gutta-percha (Ariadent, Asia Chemi Teb Co., Tehran, Iran) using backfill technique with Beefil system (VDW, Munich, Germany) and then the teeth were sealed with 3-4 mm dressing paste (Asia Chemi Teb Co., Tehran, Iran). All samples were stored at 37ºC and 95% humidity for 1 week. For two-steps groups a moist cotton pellet was placed over the MTA and the teeth were sealed with 3-4 mm dressing paste. The MTA was allowed to set at 37°C and 95% humidity for 3 days and the remaining canal space was obturated as in the one-step groups. The samples were kept at 37°C and 95% humidity for 1 week. Two positive control samples were obturated completely with a single cone of gutta-percha without sealer and two negative control samples were obturated as the experimental groups. The samples were coated with double layer of nail varnish except for the apical 1 mm. Negative controls were entirely coated in two layers of nail varnish and sticky wax (Kerr, Berlin, Germany). Positive controls were coated as the experimental groups.

**Leakage study**

Twelve vials with snap on plastic caps (Hayyan co., Tehran, Iran) used to suspend the prepared teeth in phenol red lactose broth (Merck, Darmstadt, Germany). A hole was made through the center of every cap. Each tooth was placed into the hole of the cap up to its cementoenamel junction, and all margins sealed with sticky wax covered with two layers of nail varnish. Securing each tooth to the vial cap in this manner allowed the 2 mm of the tooth to be outside the vial. The prepared teeth along with their caps and vials were sterilized with ethylene oxide gas for 12 hours. Sterile phenol red broth with 3% lactose was placed in each vial to a level of 2 to 3 mm above the resected root end. To ensure sterilization, the whole system was incubated at 37°C for 4 days.

Staphylococcus Epidermidis (SE) organism (reference laboratory/Tehran) was cultured at 37°C for 24 hours in nutrient agar (Merck, Darmstadt, Germany). Fresh bacterial suspension of (0.04 mL, 107 CFU) SE was injected into the vials using an insulin-syringe every 4-5 days. The vials were incubated at 37°C for 60 days. In this period, the color of phenol red broth in vials changed red to yellow which was the indicator of bacterial penetration. The vials which their color changed to yellow were excluded since they indicated SE penetration and growth. To confirm the purity of SE in the inoculums, a sample was taken in these vials and was cultivated at 37°C. The organisms were identified by morphology and gram reaction at ×100 magnification. The data were statistically analyzed using the Chisquare (χ^2^) test. A value of P<0.05 was statistically significant.

## Results

**Figure 1:**
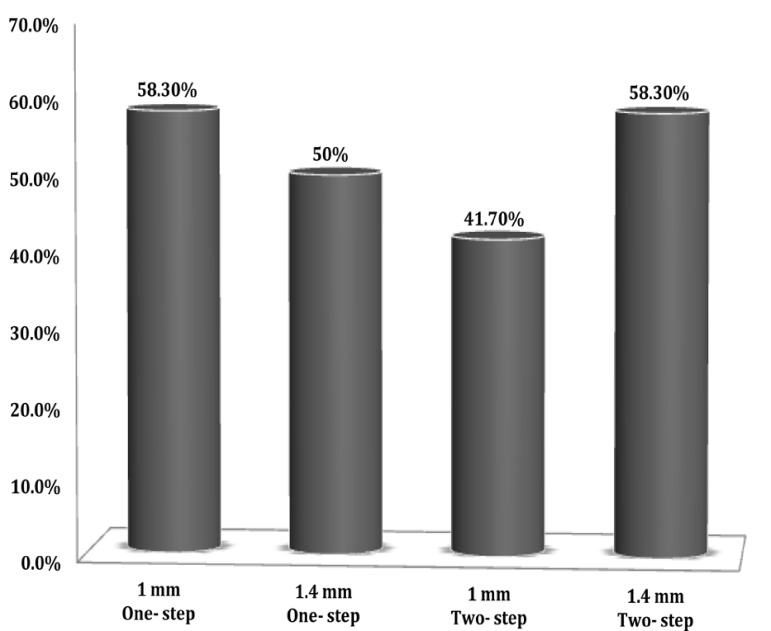
Bacterial leakage results: one-step vs. two-step technique and 1 mm vs. 1.4 mm apical diameter

There was no statistically significant difference observed between one- and two-step techniques (χ^2^ test; P=1) (Figure 1). Same results were observed where 1 and 1.4 mm apical foramen diameters were used in samples (χ2 test; P=1) (Figure 1). Results showed that the maximum microleakage occurs during the first 10 days (P=0.012).

## Discussion

We evaluated the effect of one- or two-step MTA barrier technique on apical leakage in this study by microbial leakage. When designing a study on microleakage, inadequacies in dye leakage studies must be considered. The molecular size of dye particles is less than that of bacteria and most studies measure the degree of leakage in one plane only [25]. PH and chemical reactivity may also influence the degree of dye penetration. Ahlberg et al. reported discrepancies in leakage patterns of methylene blue and India ink in root-filled teeth [26]. They found a higher level and wider variation of methylene blue penetration compared with that of India ink in all experimental groups. Similarly, in studies using radioisotope techniques, results may be affected by the type of isotope, the distance between radiation source and emulsion, and different exposure times. Furthermore, radioisotope tracers are smaller than bacteria and distribute differently [25]. Because of a lack of correlation between dye particles, isotopes and bacterial microleakage, the efficacy of root-end filling materials may be better determined using a bacterial microleakage model [27].

The results showed no statistical difference between one- and two-step techniques. In addition, the results didn’t show any statistical difference between 1 and 1.4 mm apical foramen diameter. Despite of our results, Matt et al. demonstrated that the two-step technique showed significantly less leakage than the one-step technique of barrier placement and concluded that additional moisture from a cotton pellet is crucial for MTA to establish its optimum properties [23]. This difference could be explained by different methods of evaluating the leakage, different time intervals and different thickness of MTA plug in studies. AL-Kahtani et al. demonstrated that two-step application failed to provide adequate sealing that is in agreement with our study [22]. It is likely that MTA plug lacks cohesion and does not set flat or sets with irregularities [22]. Based on our experimental conditions, 52.1% of the MTA barriers demonstrated bacterial leakage. The maximum microleakage occurred in the first 10 days. It takes time for MTA to absorb humidity and to obtain its optimal properties [28]. Hachmeister et al. showed that 91% of the MTA barriers demonstrated bacterial leakage by day 10 which is in agreement with our findings [29].

We did not find any similar study to compare the effect of apical diameter with our study. Evaluating the effect of apical diameter on leakage of 4 mm MTA plug and its probable effects on humidity absorption of MTA were reasons for selecting two different diameters. In this study, the samples with 1.4 mm apical diameter intended to show more leakage than the samples with 1 mm apical diameter but it was not statistically significant. It is concluded that the 4 mm thickness of MTA plug in both 1 and 1.4 mm diameter can seal the apical area. Aminoshariae et al. showed that there was no significant difference in the results for any of the four lengths of 3, 5, 7, and 10 mm of MTA plug placed by the hand method [30]. It seems that the minimal MTA thickness for adaptation to the dentinal walls is 3 mm. The results of different apical diameters may be much more apparent if less MTA thickness was selected. It seems that 0.4 mm difference in plug thickness will not result in significant leakage differences. In addition, occurred leakage in small-diameter plug can be attributed to less leakage surface and better condensation, and that of thicker plug can be related to its more humidity absorption. Orthograde delivery of material is technique sensitive. Placement must be verified with radiographs and condensation is limited because of minimal resistance of open apex. In addition to the difficulty in delivering the material to the apex, the irregularities and divergent nature of the anatomy may limit adaptation to the dentinal walls, creating marginal gaps at the dentinal interface [22][29].

## Conclusion

Considering the limitations of this in vitro study, it is concluded that MTA plug can be placed in one-step barrier technique because of two-step technique disadvantages i.e. tooth fracture and loss of coronal seal. More in vivo experiments are needed for investigating clinical use of one-step techniques. Future clinical studies focused on different apical diameters and different thicknesses of MTA as an apical barrier would lend further support to this treatment option.
